# Capillary hemangioma involving both intracranial and entire spinal dura: a case report

**DOI:** 10.3389/fonc.2026.1744723

**Published:** 2026-05-08

**Authors:** Zixuan Wei, Jibo Zhang, Huan Li, Chengshi Xu, Jincao Chen

**Affiliations:** 1 Department of Neurosurgery, Zhongnan Hospital of Wuhan University, Wuhan, China; 2 Brain Research Center, Zhongnan Hospital of Wuhan University, Wuhan, China; 3 Department of Radiology, Zhongnan Hospital of Wuhan University, Wuhan, China

**Keywords:** capillary hemangioma, case report, intracranial, radiotheapy, whole spinal dural

## Abstract

**Background:**

Capillary hemangiomas of the central nervous system are exceedingly rare. Previously documented cases have predominantly been located in the intracranial or spinal regions, with the majority presenting as solitary focal lesions.

**Case description:**

A 57-year-old woman complained back pain, urinary problems, and numbness and weakness in the lower extremities. MRI revealed thickening and enhancement of the entire spinal dura mater, anomalous enhancement signals surrounding cerebral blood vessels, and DSA revealed multiple malformed vascular clusters in the intracranial and spinal cord regions. Subsequently, a dural biopsy confirmed the pathological diagnosis of capillary hemangioma, with further whole-genome sequencing to identify potential gene mutations.

**Conclusions:**

Central nervous system capillary hemangiomas can affect the entire spinal cord and intracranial meningeal tissues simultaneously. Genetic alterations, including PDE4DIP mutations, were identified, but their clinical significance remains unclear.

## Introduction

Capillary hemangiomas are an expanded capillary network commonly found in infants’ skin or soft tissues, especially on the face, scalp, and chest/back (1, 2). Cases of central nervous system capillary hemangiomas include intracranial, spinal epidural, and spinal subdural types. Intracranial capillary hemangiomas mostly originate from the dura mater, with a few cases of intraparenchymal hemangiomas reported (3). Spinal epidural hemangiomas have 55 reported cases (4), and spinal subdural hemangiomas have 25 reported cases, mostly limited to 1–2 spinal segments.

This article presents a rare case of central nervous system capillary hemangioma involving both the intracranial and entire spinal cord segments. It includes a detailed description of the clinical history, examinations, diagnosis, treatment process, histopathological indicators, and prognosis of the case.

## Case description

A 57-year-old East Asian woman presented with a >2-month history of back pain, initially tolerable, followed by 20 days of progressive numbness in both lower limbs, the ulnar side of the right hand and forearm, lower limb weakness, and autonomic dysfunction, including urinary retention and constipation. As symptoms gradually worsened, she sought care at a local hospital. Initial non-contrast MRI showed no obvious space-occupying lesion. Based on the clinical presentation, central nervous system inflammation was suspected, and a lumbar puncture was attempted but yielded no cerebrospinal fluid. Subsequent contrast-enhanced cervical MRI raised suspicion of a vascular malformation, and the patient was referred to our institution for further evaluation. The patient had a history of left cerebellar hemangioblastoma resection 5 years earlier and ventriculoperitoneal shunt placement for hydrocephalus 1 year prior, as well as a 20-year smoking history.

The patient is alert, oriented, with normal muscle tone in all four limbs. Muscle strength is at level IV in the lower limbs and level V in the upper limbs. Sensation in the limbs and trunk is normal and symmetrical, with physiological reflexes present and no pathological signs. No obvious perineal sensory complaints were reported by the patient; however, a formal assessment of perineal sensation was not performed. In addition, a digital rectal examination was not conducted, and objective evaluation of bladder function was not performed. Therefore, sacral nerve function, including saddle anesthesia and anal sphincter tone, could not be definitively assessed.

**Figure 1:**
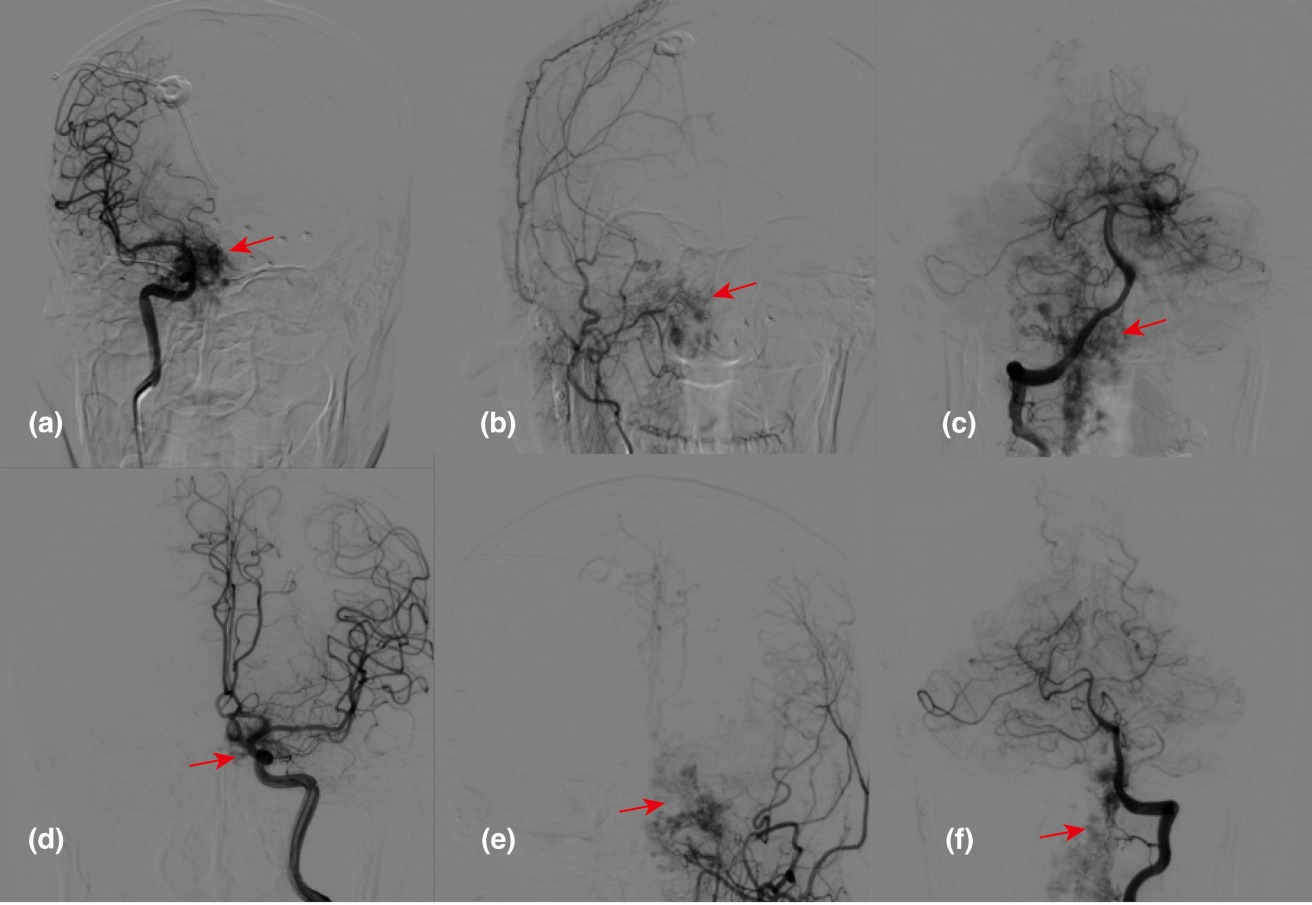
Cerebral DSA of patient indicating multiple vascular clusters. Abnormal vascular clusters (indicated by red arrows) were identified in the bilateral internal carotid arteries **(a, d)**, external carotid arteries **(b, e)**, and vertebral arteries **(c, f)** during angiography.

After admission, cerebral digital subtraction angiography (DSA) was initially performed, as prior MRI studies at the local hospital had already suggested a possible vascular malformation. However, intra-procedural findings indicated various vascular malformations in the internal carotid arteries, external carotid arteries, and vertebral arteries, indicating potential inflammatory vasculopathy (**Figure 1**). Subsequent spinal angiography revealed multiple segmental malformations, but the exact disease type remained undetermined (**Supplementary Figure 2**).

**Figure 2:**
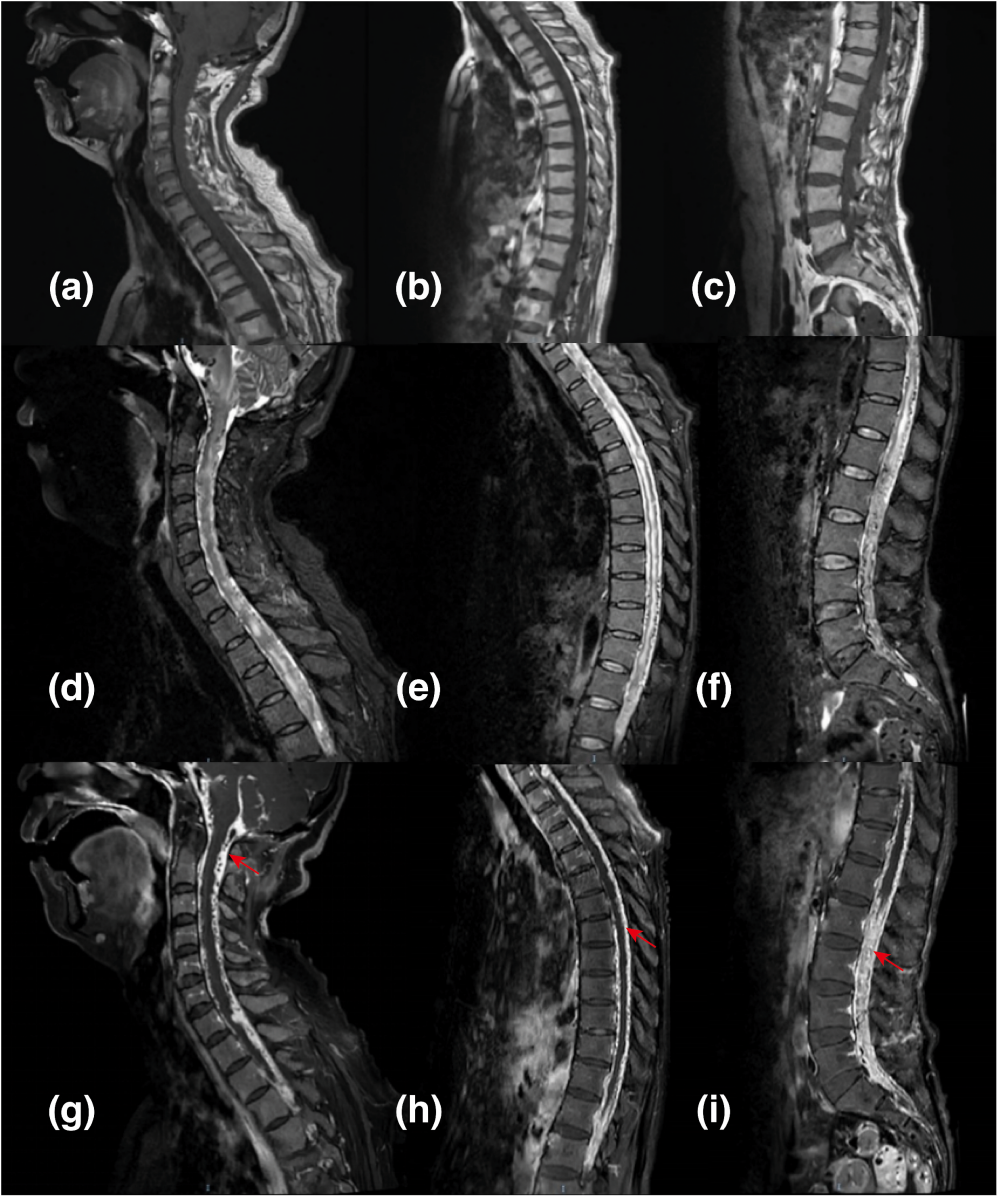
Spinal MRI before biopsy. Strip-like shadows with long T1 **(a–c)** and T2 **(d–f)** signals are visible throughout the spinal cord, without enhancement **(g–i)**. The dura mater surrounding the spinal cord shows significant uneven thickening, appearing as long T1 **(a–c)** and T2 **(d–f)** signal shadows, with uneven enhancement [**(g–i)**, red arrows].

**Figure 3:**
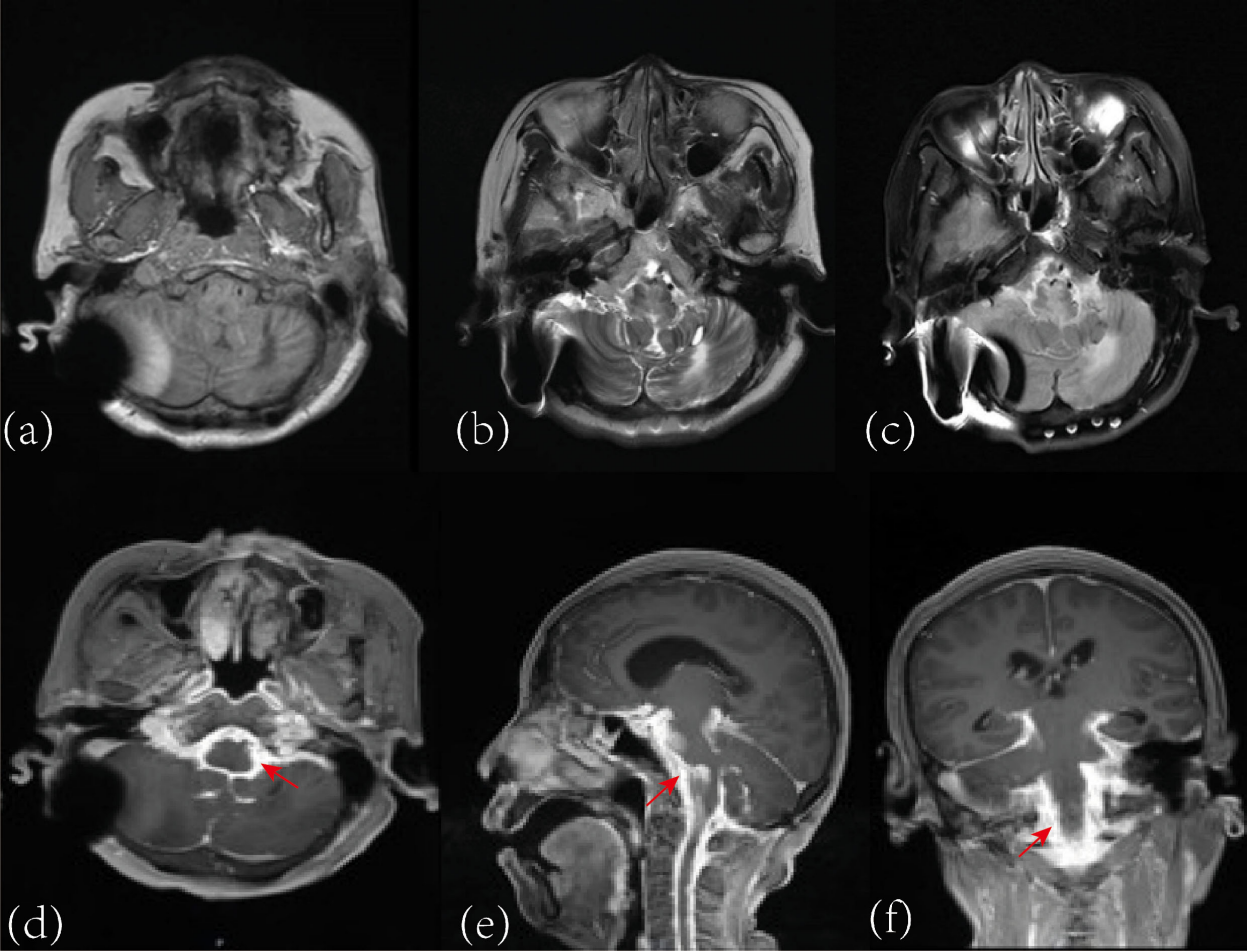
Brain MRI suggesting abnormal signals surrounding major arteries and some veins, potentially blocked the vascular. The plain scan sequence reveals patchy and linear long T1 **(a)** and long T2 signal **(b)** shadows in the bilateral cerebral arterial flow regions. High signal intensity is observed in the Flair sequence **(c)**. The enhanced sequence displays multiple linear and patchy enhancement shadows surrounding the bilateral internal carotid arteries, vertebral arteries, cerebral arteries, and some veins [**(d–f)**, red arrows]. The signal artifact in the right cerebellar region is caused by the ventriculoperitoneal shunt device placed in the right lateral ventricle.

For further evaluation, subsequent MRI of the brain and entire spinal cord was then performed at our institution. It revealed strip-like long T1 and long T2 signal shadows within the spinal cord, with uneven thickening of the dura mater. Enhanced MRI reveals multiple linear and strip-like enhancement shadows around the bilateral internal carotid arteries, vertebral arteries, and some veins, with some arterial lumen narrowing or occlusion. The thickened dura mater around the spinal cord shows significant uneven enhancement, without other obvious abnormal enhancements within the spinal cord (**Figures 2**, **3**). The radiologist suggested the possibility of dural/meningeal arteriovenous fistula with spinal cord ischemic changes.

**Figure 4:**
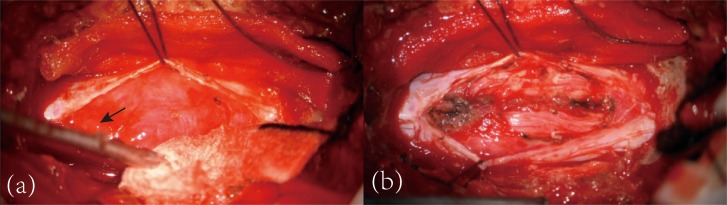
Tumor exposure and excision. After cutting the dura mater, a red, soft, and well-vascularized mass was found on the surface of the spinal cord [**(a)**, black arrow] and then dissected along its surface **(b)**.

In order to identify the lesion, a dural biopsy was performed under general anesthesia. The procedure focused on the L1–2 area using a posterior midline approach. The spinal cord tension was normal, and clear cerebrospinal fluid was observed upon incision. A visible, red, soft mass with a rich blood supply was found on the spinal cord surface. The mass was carefully removed and sent for examination (**Figure 4**).

The microscopic examination diagnosed it as a capillary hemangioma (**Supplementary Figure 3**). Immunohistochemical testing of the tumor cells showed: CD34(+), Ki-67(Li: 3%), ERG(+), а-inhibin(weak+), NSE(-), CAIX(-), GFAP(-).

The results of whole-genome sequencing reveal pathogenic mutations in the exonic regions of PERM1 and PDE4DIP, with a suspicious mutation in ITPKB.

After 15 days post-surgery, the patient started skull base and whole spinal cord TOMO radiotherapy (PTV-B=19.5Gy/13F). Concurrently, she received intravenous dexamethasone 5mg twice daily and 125ml of mannitol every 8 hours to reduce edema post-radiotherapy. 33 days post-surgery, the patient was discharged but experienced breathing difficulties and loss of appetite at home, they did not seek medical attention. Unfortunately, the patient passed away at home 5 days later.

## Discussion

Capillary hemangioma is a benign tumor commonly found in infants and young children, primarily on the skin (5). It typically appears shortly after birth, grows rapidly within 6–9 months, and then gradually regresses, usually disappearing before age 10 (6, 7). While it can also occur in internal organs like the lungs, liver, and kidneys, occurrences in the central nervous system are rare (8–10). Reported locations in the central nervous system include the brain, dura mater, spinal epidural, and spinal subdural, with most cases being focal lesions. Spinal cord lesions are usually limited to 1–2 segments, with very few cases involving 3 segments or having 2 isolated spinal cord lesions. There are no reported cases of capillary hemangioma affecting the entire spinal cord segment or simultaneously impacting the brain and spinal cord.

In central nervous system capillary hemangiomas, the most common symptom in patients with lesions inside the skull is headache (40%) and cranial nerve paralysis (30%) (11). For patients with lesions in the spinal cord, pain (60%) and limb weakness are most common (4). In the case discussed, the patient initially had back pain, followed by numbness, limb weakness, and difficulty urinating. Magnetic resonance imaging shows strip-like regions with prolonged T1 and T2 signals in the spinal cord and increased intensity on fat suppression sequences, suggesting possible spinal cord ischemia. During surgery, a tumor on the spinal cord’s surface was observed, likely causing the patient’s symptoms due to compression or invasion of the lesion leading to spinal cord dysfunction.

The diagnosis of central nervous system capillary hemangioma relies on histopathological features and immunohistochemical examination. Imaging studies may show certain characteristics, but a definitive diagnosis cannot be based solely on imaging. Central nervous system capillary hemangiomas typically present as focal lesions with isointensity on T1-weighted images, hyperintensity on T2-weighted images, and significant enhancement on contrast-enhanced images (12). Some cases may also show flow voids within the lesion on T2 sequences (11). In this case, the imaging findings similarly showed significant enhancement of the dura mater and thecal sac, with multiple flow voids within the thecal sac vessels. DSA revealed malformed vascular clusters in the intracranial major arteries and throughout the spinal cord. While it is challenging to differentiate whether these were inflammatory vessels, this provided a basis for distinguishing from dural arteriovenous fistulas. Histopathological examination of the tumor tissue in this case showed abundant proliferating capillaries with lobular structures. Immunohistochemistry indicated positivity for features consistent with capillary hemangiomas reported in the literature, such as CD34(+), supporting the final diagnosis (13). Given the diffuse central nervous system involvement, careful differential diagnosis is essential. Spinal dural arteriovenous fistula may present with progressive myelopathy and vascular abnormalities; however, typical angiographic features such as arteriovenous shunting and characteristic venous drainage were not observed. Inflammatory conditions, such as myelitis, may show similar clinical features but usually differ in imaging and lack vascular proliferation on histopathology. Overall, the combined histopathological and immunohistochemical findings favor a diagnosis most consistent with capillary hemangioma.

Central nervous system capillary hemangiomas are rare, and it is unclear if they are linked to genetic factors. However, gene mutations have been associated with non-central nervous system capillary hemangiomas. For instance, mutations in the EIF2AK4 gene are linked to pulmonary capillary hemangiomatosis, while mutations like p.Glu70Lys and p.Trp88Ter may be connected to retinal capillary hemangiomas (14, 15). In infantile capillary hemangiomas, genes with abnormal expression during disease progression, such as GLUT-1, VEGF, and MMP9, have been identified (16). Whole-genome sequencing was conducted on central nervous system capillary hemangioma tissue for the first time to investigate the causes of its occurrence and extensive lesions. The results revealed clinical pathological mutations or suspected clinical pathological mutation genes in three exonic regions (PERM1, PDE4DIP, ITPKB). PERM1 (PGC-1/ERR-induced regulator in muscle 1) is involved in the mitochondrial function of cardiac or skeletal muscle cells (17). PDE4DIP (Phosphodiesterase 4D interacting protein)interacts with cAMP-specific phosphodiesterase 4D, and its abnormal expression promotes the development of hematologic malignancies, breast cancer, and pineal cell tumors (18). Mutations have been detected in various malignant tumors like hepatocellular carcinoma, thyroid cancer, and gliomas (19–21). ITPKB (inositol-trisphosphate 3-kinase B)is crucial for the development, survival, and function of B cells, with related mutations reported in Hodgkin’s lymphoma and diffuse large B-cell lymphoma (22, 23). Although these genes have not been previously linked to capillary hemangiomas, the identified mutations may suggest a potential association with the extensive lesions. However, this observation is exploratory and requires further validation.

In previous research, central nervous system capillary hemangiomas have been primarily treated through surgical resection (3, 4). This treatment has shown to relieve neurological function disorders in 55-66% of cases, with no recurrences in cases of complete resection. Radiation therapy is used for cases where complete surgical resection is not possible, such as when the tumor is located in challenging areas like the cavernous sinus or Meckel’s cave (24). While there are no reported cases of radiation therapy for spinal cord capillary hemangiomas, stereotactic radiotherapy is considered a safe and effective treatment for hemangioblastoma (25). However, evidence supporting the use of radiotherapy in diffuse spinal cord involvement remains extremely limited. Studies have also explored the use of oral propranolol in treating infantile brain or spinal cord capillary hemangiomas (26). Considering the diffuse involvement of both intracranial and entire spinal meningeal structures, complete surgical resection was not feasible, and radiotherapy was therefore selected based on prior experience with similar vascular tumors. In this case, craniospinal irradiation was applied to achieve broad coverage of the neuraxis. However, the optimal radiotherapy strategy for such extensive lesions remains unclear. Large-field irradiation may increase the risk of treatment-related neurotoxicity, particularly in patients with pre-existing spinal cord dysfunction. The patient developed symptoms such as dyspnea and poor appetite after discharge and unfortunately passed away shortly thereafter. While rapid disease progression cannot be excluded, the short-term deterioration may have been associated with radiation-induced edema or further impairment of spinal cord function. Therefore, this case highlights that radiotherapy should not be considered a standard approach for such diffuse conditions, and its use requires careful risk–benefit assessment. Alternative strategies, including staged or segmental radiotherapy, dose optimization, and intensive supportive management, may be considered to reduce potential complications. Further studies are needed to establish safer and more effective treatment strategies.

In summary, this case enhances our understanding of capillary hemangiomas in the central nervous system, demonstrating their significant impact on the spinal cord and intracranial regions simultaneously. Imaging characteristics were consistent with previously reported cases of spinal cord capillary hemangiomas, showing prolonged T1 and T2 relaxation times and marked enhancement. Meanwhile, the patient’s clinical manifestations resembled spinal cord compression. The definitive diagnosis was also supported by pathological findings that demonstrated extensive capillary proliferation exhibiting a lobular architecture, in conjunction with immunohistochemical analysis revealing CD34 positivity. However, this case is not without its limitations. Firstly, the management of the patient’s condition post-discharge was neither timely nor effective, ultimately resulting in the patient’s demise, which hinders our ability to assess the long-term efficacy and prognosis following treatment. Secondly, while we identified potential tumor development-related genes through whole-genome sequencing, it is important to note that this remains a case report and may be subject to inherent biases. Meanwhile, a comprehensive evaluation of sacral nerve function, including perineal sensation, anal sphincter tone, and bladder function, was not performed, which limited the assessment of possible cauda equina or conus medullaris syndrome.

## Conclusion

Central nervous system capillary hemangiomas can affect the entire spinal cord and intracranial meningeal tissues simultaneously. The underlying mechanisms remain unclear, and genetic findings, including PDE4DIP mutations, require further validation. It must be distinguished from other conditions like central nervous system inflammation and spinal arteriovenous fistula. Diagnosis is based on histology. Radiotherapy of the whole spinal cord and skull base can help relieve symptoms of spinal cord compression, but long-term clinical management is essential. Prompt treatment of complications can improve the prognosis.

## Data Availability

The original contributions presented in the study are included in the article/**Supplementary Material**, further inquiries can be directed to the corresponding author.
