# Supplementary material for: Capillary hemangioma involving both intracranial and entire spinal dura: a case report
Source: Front Oncol. 2026 May 8;16:1744723. doi: 10.3389/fonc.2026.1744723 (PMC13194041; doi:10.3389/fonc.2026.1744723)
Supplement: Supplementary file 1 [file DataSheet1.docx]

**
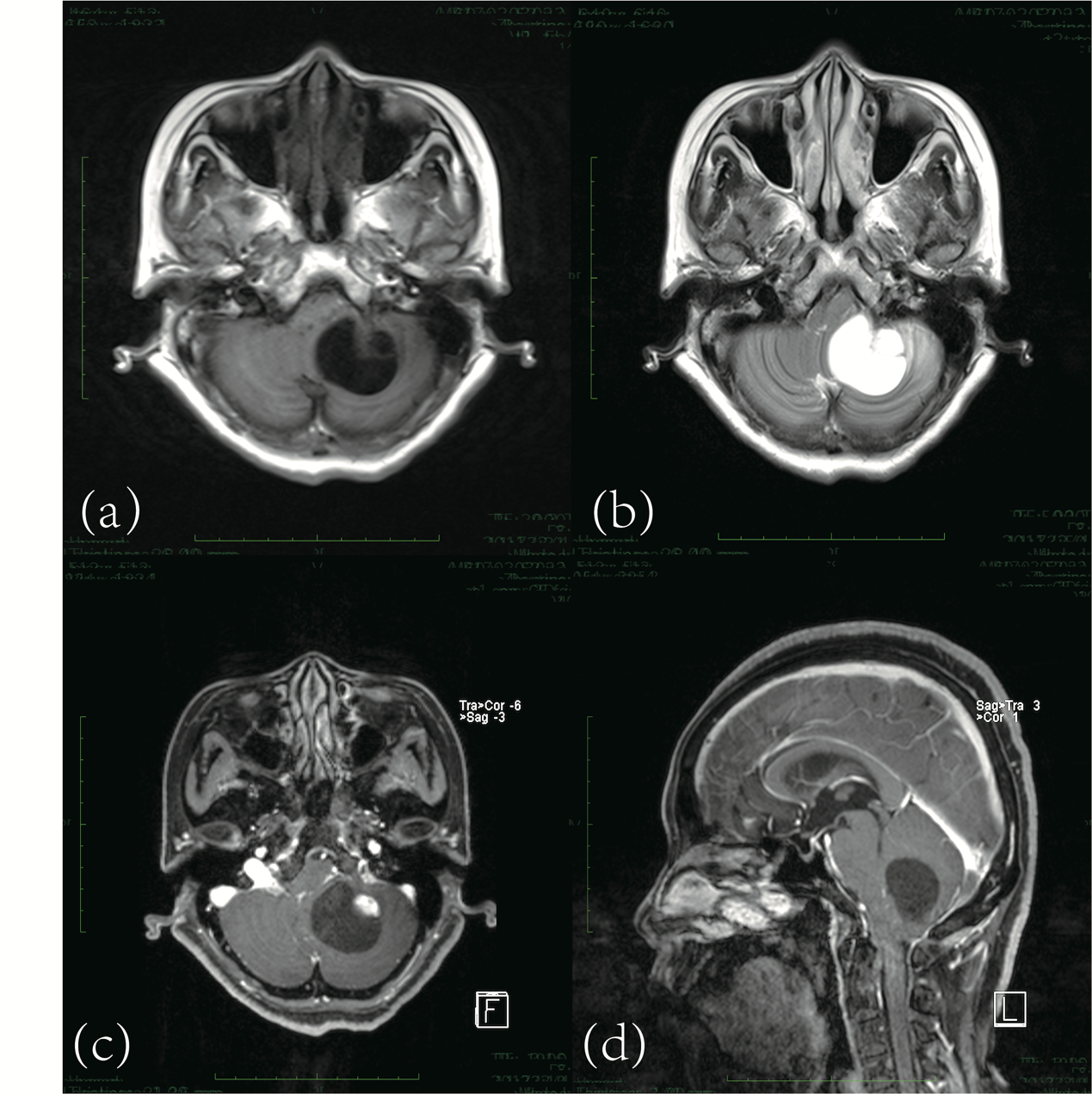
**

**Figure S1** Preoperative MRI performed 5 years ago suggesting the left cerebellar mass. The tumor has a rounded morphology, hypointensity on T1WI (a), and hyperintensity on T2WI (b), with well-defined margins. Small nodular shadows within the tumor show marked enhancement during the enhancement sequence (c), while the surrounding tissue shows no significant enhancement (d).


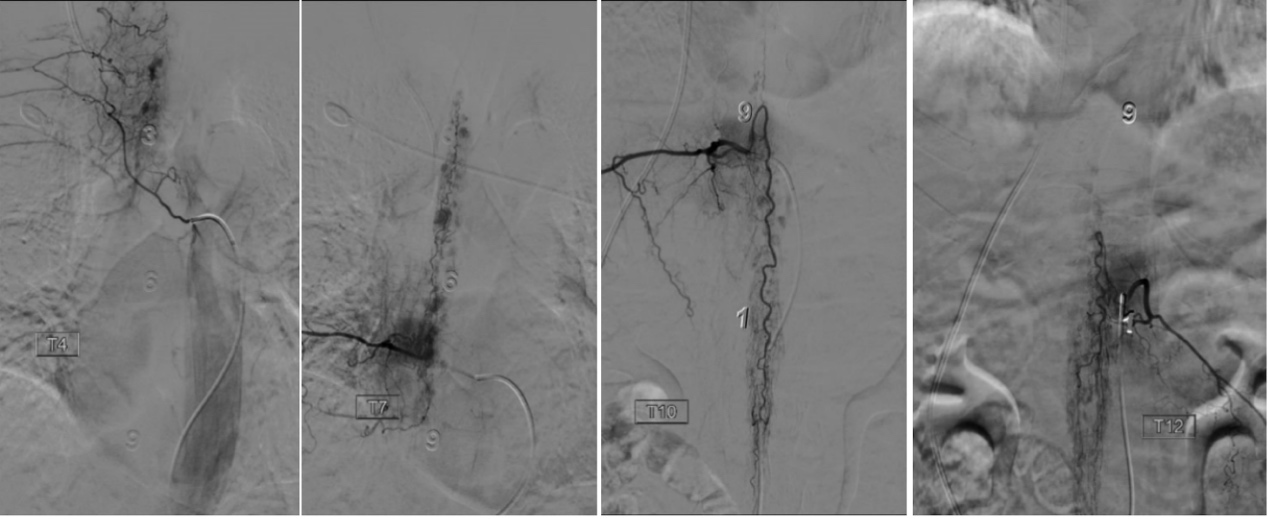


**Figure S2** Spinal DSA revealed multiple cloud-like abnormal vascular clusters similar to those in the brain.


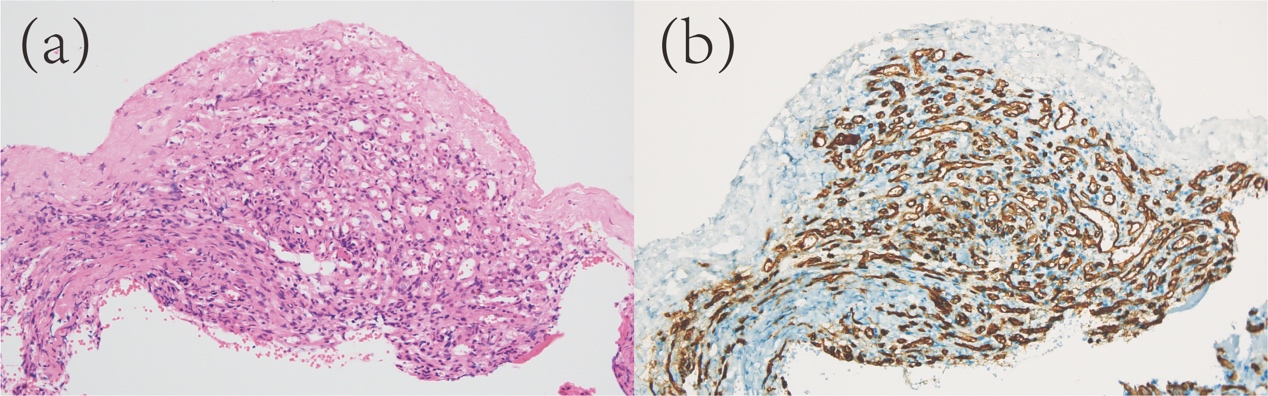


**Figure S3** Pathological section of surgical specimen. A densely populated vascular structure resembling numerous capillaries, characterized by the presence of endothelial cells and hemosiderin deposition, was observed, indicating a strong likelihood of capillary hemangioma. (a) original magnification ×100, (b) IHC of CD34(+).
